# Caveolae and Caveolin-1 Integrate Reverse Cholesterol Transport and Inflammation in Atherosclerosis

**DOI:** 10.3390/ijms17030429

**Published:** 2016-03-22

**Authors:** Li Qin, Neng Zhu, Bao-Xue Ao, Chan Liu, Ya-Ning Shi, Ke Du, Jian-Xiong Chen, Xi-Long Zheng, Duan-Fang Liao

**Affiliations:** 1 School of Pharmacy, Hunan University of Chinese Medicine, Changsha 410208, China; lqin1011@126.com (L.Q.); aobaoxue@126.com (B.-X.A.); iriniliu@163.com (C.L.); syndyx0808@163.com (Y.-N.S.); Cocoadu006@163.com (K.D.); jchen3@umc.edu (J.-X.C.); xlzheng@ucalgary.ca (X.-L.Z.); 2 Department of Urology, The First Hospital of Hunan University of Chinese Medicine, Changsha 410208, China; Nzhu0116@126.com; 3 Department of Pharmacology and Toxicology, University of Mississippi Medical Center, School of Medicine, Jackson, MS 39216, USA; 4 Department of Biochemistry & Molecular Biology, the Libin Cardiovascular Institute of Alberta, University of Calgary, 3330 Hospital Drive NW, Calgary, AB T2N 4N1, Canada

**Keywords:** caveolae, caveolin-1, reverse cholesterol transport, inflammation, atherosclerosis

## Abstract

Lipid disorder and inflammation play critical roles in the development of atherosclerosis. Reverse cholesterol transport is a key event in lipid metabolism. Caveolae and caveolin-1 are in the center stage of cholesterol transportation and inflammation in macrophages. Here, we propose that reverse cholesterol transport and inflammation in atherosclerosis can be integrated by caveolae and caveolin-1.

## 1. Introduction

Atherosclerosis (AS) has been identified as a systemic, inflammatory disease of the arterial wall. Accumulation of cholesterol ester in the arterial wall promotes the formation of atherosclerotic plaque, thickening, and stenosis of the vessel wall, leading to subsequent cardiovascular and cerebrovascular diseases. During the pathological process, macrophages derived from monocytes, vascular smooth muscle cells (VSMCs) and endothelial cells are transformed into lipid-loaded cells via uptake of lipoproteins, especially oxidized low-density lipoprotein (ox-LDL). The capacity of reverse cholesterol transportation (RCT) in lipid-loaded cells is a key factor to determine the outcome of AS. Generally, RCT is considered as the main pathway by which accumulated cholesterol is transported from the vessel wall to the liver for excretion, and a transintestinal cholesterol efflux as compensation [1], thus preventing AS. Here, we focus on the role of RCT involving cholesterol efflux from cells. Based on exciting findings from other investigators and our laboratory, we proposed a novel model of cholesterol transportation, including one center and four systems with coupling transportation and networking regulation [2]. This novel model consists of a caveolae transport center, an intracellular trafficking system of the caveolin (Cav)-1 complex, a transmembrane transport system of the ATP-binding membrane cassette transporter A1 (ABCA1) complex and the scavenger receptor class B1 (SR-B1) complex, and an extracellular trafficking system of high-density lipoprotein (HDL)/apolipoprotein A1 (apo-A1). It is believed that macrophages and VSMCs are designated as lipid-loaded cells if the ratio of cholesterol esters in the total cholesterol is less than 50% in these cells, and are considered as foam cells if the ratio is more than 50%. Most importantly, lipid-loaded cells have strong RCT capability (at the earlier stage), whereas foam cells have very weak or no RCT capability [2].

In 1999, Ross first proposed the inflammation hypothesis of AS [3]. He have pointed out that lipid-loaded cells release many inflammatory cytokines and proteins to mediate non-specific immune responses, promoting the progression of AS. That is to say that AS has the characteristics of chronic inflammatory response. Many inflammatory cells and inflammatory mediators are involved in the pathogenesis of AS, from the plaque formation to plaque rupture, as well as various stages of thrombosis.

Recent studies revealed that inflammation may impair RCT in the process of AS [4,5,6]. However, the molecular mechanism between inflammation and RCT remains largely unknown. In this review, we focus on caveolae and Cav-1, as a key player in modulating inflammation and RCT through their direct and indirect anti-inflammatory mechanisms.

## 2. Relationship between RCT and Inflammatory Response

### 2.1. Effect of RCT on Inflammatory Response

Accumulating evidence suggests that cholesterol itself is an inflammatory factor. Cholesterol, when over-loaded into the extracellular space, creates an inflammatory environment. In the absence of other inflammatory stimulation, excessive free cholesterol within the membrane and/or endosomes can activate the p38 mitogen-activated protein kinase (MAPK) signaling pathway via toll-like receptor (TLR) 3 or TLR4, and then induces p38 target genes, including cathepsin K (CTSK), S100 calcium binding protein A8 (S100A8), matrix metalloproteases (MMP)8, and MMP14, and potentially contributes to plaque complications, such as aneurysm formation and plaque disruption [7]. By moving intercellular adhesion molecule 1 (ICAM-1) out of caveolae, cholesterol increases adhesion of monocytes to endothelium [8], and depletion of cholesterol by cyclodextrin inhibits interleukin (IL)-1β-related inflammation [9].

Ox-LDL, a pro-atherogenic particle, is also considered to be an inflammatory factor. It has been implicated in modulating the expression of several cytokines, growth factors and lipopolysaccharide (LPS)-induced inflammatory cytokines that are regulated by the nuclear factor κ-light-chain-enhancer of activated B cells (NF-κB) pathway. Oxidation of LDL could be triggered by aggregatibacter actinomycetemcomitans-induced inflammation [10]. Statins (or HMG-CoA reductase inhibitors), as a class of drugs used to lower cholesterol levels, could disrupt primary receptor of ox-LDL or lectin-like oxidized low-density lipoprotein (LDL) receptor-1 (LOX-1) and alleviate ox-LDL functions [11].

Remnant lipoproteins (RLPs) may have a causative role in inflammation by up-regulating endothelial expression of ICAM-1, vascular cell adhesion molecule 1 (VCAM-1), and tissue factor (TF) [12]. In children with obesity, remnant lipoproteins (RLPs) could be an effective biomarker for pediatric cardiovascular disease (CVD) risk [13].

HDL is thought to be an important anti-inflammatory particle, which plays a primary role in mediating RCT and cholesterol efflux through the influence of paraoxonase/arylesterase 1 (PON1), IL-8, monocyte chemotactic protein (MCP), nitric oxide (NO), tumor necrosis factor (TNF)-α, triphosphopyridine nucleotide (NAPDH), VCAM-1 and ICAM-1 [14]. HDL promotes RCT through apoA-1, in which small HDL particles promote cholesterol efflux through ABCA1, and large particles through SR-B1 and ATP-binding membrane cassette transporter G1 (ABCG1) [15]. SR-B1 that binds with HDL mediates many anti-inflammatory and anti-apoptotic effects via initiation of various downstream signaling pathways involving activation of both protein kinase B (Akt) and MAPKs and eventually activation of endothelial nitric-oxide synthase (eNOS). However, oxidation of HDL generates a particle that loses its anti-inflammatory activity, but gains a pro-inflammatory function, as suggested by up-regulation of endothelial cell VCAM-1 protein and the activation of nuclear factor κ-light-chain-enhancer of activated B cells (NF-κB) via the IKKs–IkB kinase (IKK) complex [16]. A recent study showed that modified LDL lost the affinity of eNOS with SR-B1, which impairs their protective role and deteriorates the situation [17]. Under the inflammatory condition, the composition of HDL is altered, and cholesterol efflux associated with SR-B1 and ABCA1 is impaired [18]. In patients with active rheumatoid arthritis, for example, HDL has a declined capacity to induce RCT. The ability of HDL to promote cholesterol efflux is positively related to its antioxidant capacity [19].

Apo-A1, the main protein component of HDL particles, exerts anti-inflammatory effects by promoting cholesterol efflux via ABCG1 and ABCA1 with consequent attenuation of the signal transduction via Toll-like receptors [20]. Our study also found that ABCA1 as a major receptor of apo-A1 in macrophages had protective effects on inflammation in response to LPS, palmitate and cytokines [21,22]. Zhu and colleagues [23] revealed that macrophages from macrophage-specific ABCA1 knockout mice had an increased pro-inflammatory response to the TLR4 agonist and LPS when compared with the wild-type mice. Notably, more and more TLR4 was found in membrane lipid rafts in ABCA1 knockout macrophages, suggesting that ABCA1 regulates macrophage reactivity to TLR agonists by modulation of lipid raft cholesterol and TLR mobilization to lipid rafts. TLR4 together with its cognate TLR4 ligand was transported to lipid rafts induced by LPS and palmitate in macrophages [16,22,24,25,26]. Taken together, these data suggest that macrophage ABCA1 dampens inflammation by reducing myeloid differentiation factor 88(MyD88)-dependent TLR trafficking to lipid rafts by selective reduction of free cholesterol contents in lipid rafts.

### 2.2. Effect of Inflammation on RCT

RCT is a multi-step and complicated process. A critical part of RCT is cholesterol efflux, in which accumulated cholesterol is removed from cells in the subintima of the vessel wall to HDL or Apo-A1 by a variety of mechanisms, including ABCA1, ABCG1, SR-B1 and Cav-1 [27]. Reduction of RCT has been observed in an inflammatory animal model induced by LPS [28]. Some studies showed that LPS inhibits cholesterol efflux in macrophages through down-regulating ABCA1 and SR-B1 expression [29]. In the new hypercholesterolemia zebrafish model, knockdown of TLR4 can significantly reduce lipid accumulation in macrophages at the early stages of atherosclerotic plaque [30]. Additionally, LPS decreased ABCA1 expression in foam cells through the NF-κB-dependent pathway [31]. Besides, pro-inflammatory endothelial cells (ECs) dysfunction induced by LPS is associated with the deficiency of Intersectin-1s (ITSN-1s). Pro-inflammatory endocytosis and permeability dysfunction in ECs could be reversed by up-regulation of ITSN-1s [32]. In human macrophages, TNF-α inhibits RCT and enhances cholesterol uptake via the protein kinase C (PKC)-θ-dependent pathway [33]. RCT could also be disturbed by infection. The intravenous challenge with aggregatibacter actinomycetemcomitans promoted the oxidation of LDL and innate immune signaling, which accelerate the development of AS [34]. Other inflammatory factors such as zymosan and ethanol also have effects on RCT [35,36].

Annema and others reported that serum amyloid A (SAA) and myeloperoxidase (MPO) could inhibit RCT during chronic inflammation [28]. It is recognized that C-reaction protein (CRP), a fundamental marker of inflammation, is associated with an increased risk of AS. The pro-inflammatory and proatherogenic properties of CRP have been found in endothelial cells, VSMCs and monocyte-macrophages. CRP inhibits cholesterol efflux from human macrophage-derived foam cells and decreases the expression levels of ABCA1 and ABCG1 [37]. Additionally, CRP increases superoxide anion production through mitochondrial dysfunction and up-regulation of NADPH oxidase subunits. In turn, increased superoxide anion induces MAPK activation, which may function as a signal transduction pathway for the effects of CRP on macrophage-derived foam cells. Furthermore, the studies from our laboratory and others show that many inflammatory factors, such as interferon-γ (INF-γ), TNF-α, IL-1β, and IL-6, could inhibit the expression of ABCA1 and ABCG1 [21,38].

## 3. Roles of Caveolae/Caveolins in RCT and Inflammatory Response

### 3.1. Caveolae and Caveolins

Caveolae, 50–100 nm plasma membrane invaginations, were first discovered by Yamada in 1955 [39]. Caveolae are a unique form of lipid rafts. These flask-shaped structures are enriched in lipids, such as cholesterol and sphingolipids. In fact, the formation and maintenance of caveolae depend on cholesterol. Several observations support a role for caveolae in maintaining cellular cholesterol balance. One of their functions is to serve as a site of cholesterol uptake from the extracellular environment [40]. These flask-shaped lipid-raft structures have many functions in cholesterol homeostasis, cell signaling, cell proliferation and migration, and endocytosis [41]. The formation and maintenance of caveolae are primarily dependent on caveolins, the main protein component of caveolae. Caveolin family consists of multifunctional 21–24 kDa signaling proteins including Cav-1, 2 and 3. The functions of caveolins are closely related to maintaining cellular endocytosis and exocytosis of caveolae as well as the regulation of intracellular signal transduction. Cav-2 and Cav-3 are also involved in RCT and inflammation. In bone marrow-derived macrophages, for example, only CAV-2 is expressed, and its expression level was decreased after stimulation of the cells with LPS, IFN-γ, and/or hypoxia [42]. However, Cav-2 and Cav-3 are not widely expressed in various cells and not as important as Cav-1 in RCT. Their roles in RCT remain to be investigated. Cav-1, present in most cell types of the cardiovascular system, is a high-affinity lipid binding protein. The expression of Cav-1 is dramatically upregulated in aortas with perivascular adipose tissue (PVAT) transfer and involved in lipoprotein transcytosis and inflammation in human ECs [43,44]. Cav-1 knockout mice showed resistant to diet-induced obesity, and also have a dysregulation in fat, muscle, and liver metabolism [45]. Cav-1 knockout mice suffer from pulmonary fibrosis and hypertension, and cardiac hypertrophy [46]. Therefore, the role of Cav-1 in RCT and inflammation is controversial. Murata *et al.* [47] demonstrated that caveolin directly binds cholesterol with high affinity in a 1:1 ratio. It has been speculated that the high concentration of cholesterol associated with caveolae is due to the large oligomeric caveolin complexes in caveolae [40]. Cav-1 is rich in caveolae, endoplasmic reticulum and Golgi bodies, and shuttles back and forth between the cytoplasm and cellular membrane [48]. Its functions are closely correlated with its levels of tyrosine phosphorylation. Schlegel [49] reports that the N-terminal membrane attachment domain (N-MAD, residues 82–101) and the C-terminus (C-MAD, residues 135–150) of Cav-1 are sufficient to anchor caveolins to the cell membrane. The highly conserved N-MAD forms the caveolin scaffolding domain (CSD), which interacts and inactivates several intracellular signaling molecules, such as the Src tyrosine kinase, G-protein α subunit, PKC-α, protein kinase A (PKA), and H-Has, for the negative regulation of signal transduction [50].

Using isolated caveolae-enriched membranes, Zhu *et al.* [8] demonstrate that cell adhesion molecules (CAMs), including intercellular adhesion molecule-1 (ICAM-1) and vascular cell adhesion molecule-1 (VCAM-1), are co-localized with Cav-1 in caveolae. LPS up-regulated the expression of CAM and increased their co-localization. Cav-1 negatively regulates monocyte adhesion by the co-localization of CAMs in caveolae, which is inhibited by cholesterol [8]. Besides, CAMs increased by Cav-1 may be downstream of inflammatory factors such as pigment epithelium-derived factor and flotillin-1 [51,52]. These data indicate that Cav-1 serves as the molecular basis of hypercholesterolemia and inflammation in atherogenesis.

### 3.2. Caveolae/Cav-1 as a Platform of RCT

Cholesterol efflux is an important component of RCT. Cholesterol deposited in the vessel intima can be transferred to HDL and apo-A1 via ABCA1, SR-B1, caveolins and 27-hydroxylase. Then esterified HDL cholesterol is transported to the liver for secretion [53]. Caveolae, as cholesterol storage pools, mediates transmembrane cholesterol transportation, endocytosis and transcytosis of lipoprotein. Many lipid-associated receptors are localized in caveolae, such as LDL-receptor, SR-B1, and ABCA1, to mediate cholesterol efflux. In fact, caveolae formation and the expression of Cav-1 are closely related to cholesterol content of the cell membrane. Their proper alignment and modification are critical in the functions of caveolae [54,55,56]. Murata *et al.* [57] found that two proline residues at the two ends of a hydrophobic region of 33 amino acids respectively result in the N-terminal and C-terminal sequences of Cav-1 to form a hairpin structure. The hydrophobic region alone is not strong enough to result in a firm binding of Cav-1 to the cell membrane. In the normal cells, 90% of the Cav-1 proteins are located in caveolae in the plasma membrane. When newly synthesized cholesterol levels or intracellular cholesterol levels are elevated, Cav-1 is translocated to the endoplasmic reticulum, Golgi bodies, and endoplasmic reticulum or round trip shuttle between caveolae and the Golgi body to transfer intracellular cholesterol to caveolae. Then, cholesterol in the membrane can be reversely transferred to the liver through the process mediated by HDL. Our studies have shown that Cav-1 has a role in cholesterol accumulation and transcytosis when cells are incubated with ox-LDL. Other investigators found that caveolin, cyclophilin A, cyclophilin 40, and HSP56 comprise the cholesterol transport complex involved in cholesterol efflux [58,59]. Intracellular free cholesterol can be transported from Golgi network to caveolae by Cav-1-VIP21 complex [60]. In addition, the interaction between Cav-1 and Sterol carrier protein-2 (SCP-2) is involved in cholesterol transportation from the endoplasmic reticulum to the membrane [61].

Cholesterol efflux is closely related to the expressions of Cav-1 and ABCA1. For example, Cholesterol efflux is increased in HepG2 cells transfected with a Cav-1 expression plasmid DNA [8]. The ABCA1 expression is increased in endothelial cells after transfection with Cav-1 cDNA, which induces cholesterol efflux. Conversely, cholesterol efflux is reduced due to decreased ABCA1 expression after transfection of small interfering Cav-1 RNA. Immunoprecipitation assay shows that Cav-1, ABCA1 and cellular cholesterol are co-localized in the Golgi aPPARatus, cytoplasm and membrane of caveolae. Inhibitors that blockade cellular lipid transportation may disrupt the interaction between ABCA1 and Cav-1, and reduce the transportation of cholesterol to HDL [62]. In the Cav-1 knockout mice, the levels of free cholesterol in peritoneal macrophages were decreased while the cholesterol ester contents were increased. Cav-1 deletion reduces free cholesterol synthesis, resulting in an increase of the activity of acyl-coenzyme A: cholesterol acyltransferase (ACAT). ABCA1 expression was significantly decreased in macrophages from Cav-1 knockout mice compared with the control macrophages. Furthermore, cells are loaded with lipids when treated with cholesterol. In addition, the ABCA1 expression in macrophages from Cav-1 knockout mice is lower than the control group, indicating that Cav-1 is involved in regulating ABCA1-mediated cholesterol efflux [63].

As the nuclear receptors, peroxisome proliferators activated receptor (PPARγ) and liver X receptors (LXRs) play pivotal roles in cholesterol homeostasis and lipid metabolism. PPARγ activation leads to repression of scavenger receptor (SR)-A. PPARγ ligands reduce triglyceride accumulation in human macrophages that are incubated with triglyceride-rich lipoproteins. Consistent with these results, PPARγ agonists inhibit macrophage-derived foam cell formation *in vivo* [64]. PPARγ positively regulates cholesterol efflux in macrophages by enhancing the expression of CD36- and LIMPII-analogous 1 (CLA-1)/SR-B1 and ABCA1 [65,66]. In response to ligand activation, LXR acts as a heterodimer with the retinoid X receptor (RXR) and binds to specific DNA response elements in the promoter region of target genes, thus regulating their expression. LXR activation leads to induction of genes including ABCA1 and Cav-1, which are involved in the cholesterol efflux pathway in an attempt to reduce the intracellular cholesterol overload. Besides, sterol regulatory element-binding protein 1c (SREBP-1c), which promotes triglyceride (TG) synthesis, is also activated by LXR. Moreover, the coactivator thyroid hormone receptor-associated protein 80 specifically activates SREBP-1c [67]. The transcription of Cav-1 is also regulated by PPARγ and LXR. Compared with adenovirus-(Ad) GFP control, PPARγ and Cav-1 are constitutively overexpressed in PPARγ adenovirus-infected RAW264.7 cells, leading to cholesterol efflux to apo-A1. Down-regulation of Cav-1 expression by a small interfering RNA approach (Cav-1-siRNA) results in a remarkable reduction of cholesterol efflux in RAW264.7 cells. Moreover, Cav-1-siRNA RAW264.7 cells treated with Ad-PPARγ showed an increased capacity to stimulate cholesterol efflux compared with non-treated Cav-1-siRNA RAW264.7 cells. In addition, 40-week-old apolipoprotein E (apoE)-deficient mice that were fed with Western-type diet and were infected with Ad-PPARγ for four weeks show an induction of Cav-1 expression in aortic vascular endothelial cells, SMCs and macrophages, and attenuation of established atherosclerotic lesions. PPARγ can induce Cav-1 expression, enhance cholesterol efflux and attenuate AS in apoE-deficient mice [68]. Additionally, PPARγ can increase SR-B1 and ABCA1 expression to promote cholesterol efflux. Cav-1, ABCA1 and ABCG1 as well as apoE have been identified as PPARγ target genes by using pharmacological inhibitors or conditional PPARγ-deficient macrophages [69,70,71,72]. Thus, Caveolae/Cav-1 serves as the transaction axis in the RCT process.

### 3.3. Caveolae/Cav-1 as a Platform for Inflammatory Response

Recently, caveolae and Cav-1 were found involving in innate immune response to inflammation. Cav-1 directly inhibits the production and release of a variety of important inflammatory cytokines including interleukin (IL) 1β, IL-2, IL-4, IL-12, granulocyte and macrophage colony-stimulating factor (GM-CSF), TNF-α, as well as RANTES (regulated upon activation, normal T-cell expressed and secreted) [69,73,74]. Cav-1-null mice display the defects in innate immunity and inflammatory immune response and susceptibility to Salmonella enteric Serovar Typhimurium infection. Moreover, macrophages derived from Cav-1 deficient mice exhibit a remarkable inflammatory response to bacterial LPS stimulation [70,75]. Cav-1 is involved in PCB (polychlorinated biphenyls)-induced inflammatory response that is atherogenic [76,77]. Cav1(−/−)/apoE(−/−) mice exhibit a 15-fold reduction in the size of plaques which contain fewer macrophages, T cells, and neutrophils. The intravital microscopic study revealed 83% less leukocyte adhesion to the vessel wall in Cav1(−/−)/apoE(−/−) mice, which attributed to reduced expression of endothelial chemokine ligand-2 (CCL-2/MCP-1) and vascular cell adhesion molecule-1 (VCAM-1). In human umbilical vein endothelial cells (HUVECs), pigment epithelium-derived factor (PEDF) bound to Cav-1 and increased the mRNA levels of MCP-1, VCAM-1 and plasminogen activator inhibitor (PAI-1) in concentration-dependent manner, thus blocking the inflammatory and thrombogenic responses [52]. Cav-1 deficiency resulted in 57% of the increase in regulatory T cells and 4% of the decrease in CD4^+^ effector T cells in lymphoid organs. The bone marrow transplantation study revealed that Cav1(−/−)/apoE(−/−) mice receiving Cav1(+/+)/apoE(−/−) or Cav1(−/−)/apoE(−/−) bone marrow revealed 4- to 4.5-fold smaller plaques without additional phenotypic changes. These studies demonstrated that Cav-1 deficiency reduced AS by impairing leukocyte infiltration into the arterial wall and activation of regulatory T-cell response [78]. Since diet and plasma-derived nutrients may modulate the inflammatory response by interacting with and altering caveolae-associated cellular signaling [79], nutritional modulation of caveolae-mediated signaling events may provide an opportunity to ameliorate inflammatory signaling pathways in the prevention of vascular diseases, including AS. It has been proposed that caveolae could be a regulatory platform for nutritional modulation of inflammatory diseases [79]. Intriguingly, Cav-2 is suggested to play an opposite role compared with Cav-1 in inflammatory responses, and the ratio of Cav-1/Cav-2 determines the outcome of LPS stimulation through the inducible nitric oxide synthase (iNOS) signaling pathway [80].

## 4. Molecular Mechanisms Underlying Caveolae/Cav-1 Integration of RCT and Inflammatory Response

Many signaling molecules involved in inflammatory response and RCT are located in caveolae, such as LPS/TLR axis, the eNOS/NO, cyclooxygenase (COX), MKK3/p38 mitogen-activated protein kinase (MARK) and integrin signaling pathways. It is Cav-1 in Caveolae that binds TLR4, eNOS, MARK, COX and integrin, and initiates different signaling pathways to regulate inflammation factors and RCT-related genes.

### 4.1. Caveolae/Cav-1 and the TLR Signaling Pathway

LPS is the main component of gram-negative bacteria membrane that induces an inflammatory response. TLR4 at the cell surface could specifically be recognized by the LPS and bacterial fatty acid. The main physiological function of TLR4 is to act as signal transduction receptor to LPS. TLR4 binding by LPS recruits the adaptor molecule MyD88 through the Tollinterleukin-1 receptor (TIR) domain of TLR4 to initiate either myeloid differentiation factor 88 (MyD88)-dependent or MyD88-independent pathways. MyD88 recruits serine/threonine kinases IL-1R-associated kinase (IRAK)4 and IRAK1. IRAK4 then phosphorylates IRAK1, resulting in recruitment of TRAF6 to the receptor complex and activation of transforming growth factor β-activated kinase (TAK1), a member of the MAPK family. Activation of TAK1 leads to the stimulation of NF-κB, a regulator of immunity and inflammation, which in turn results in the production of an array of pro-inflammatory cytokines, chemokines, and adhesive molecules, such as IL-1, IL-6, IL-8, IL-12, TNF-α, macrophage inflammatory protein (MIP), and VCAM-1. LPS could promote TLR4 cooperation with its ligand and traffic to lipid raft region in macrophages, followed by the secretion of inflammatory cytokines, suggesting the connection between Cav-1-mediated cellular cholesterol efflux and the inflammatory response. Wang and colleagues further found that TLR4 is highly co-localized with Cav-1 in caveolae fractions of peritoneal macrophages [81]. These findings strongly suggest that regulation of TLR4 function may occur within caveolae or lipid raft microdomains. Cav-1 directly interacts with TLR4 by binding to this site and functionally suppresses TLR4 complex assembly with MyD88 or (TIR-domain-containing adaptor inducing interteron-β (TRIF). The Cav-1 binding motif in the amino acid sequence of murine TLR4 (^739^ FIQSRWCIF^747^) has been identified by mutation analysis. The interaction of Cav-1 with TLR4 is mediated by the Cav-1 binding motif in TLR4 because the W744A mutation of this binding site abolishes their interaction and reverses the inhibitory effect of Cav-1 on cytokine regulation. Some molecules also HSP facilitate Cav-1-dependent signaling transduction across the cell membrane. Flotillin-1 and Cav-1 are localized within HUVECs, and the knockdown of flotillin-1 decreased Cav-1 expression. Their interaction enables inflammatory response triggered by TLR3 [51]. Ethanol or LPS triggers TLR4 endocytosis by caveolae and clathrin-dependent pathways in astrocytes [36]. In addition, Cav-1 diminishes the LPS-induced nuclear translocation of NF-κB p65. Garrean and colleagues [82] report that NF-κB DNA binding activity was significantly reduced in the lung tissue of Cav-1 null mice, and NOS inhibitors could reverse the inhibition of NF-κB activity.

### 4.2. Caveolae/Cav-1 and the eNOS/NO Signal Pathway

NO plays an important role in host defense and inflammation in addition to its regulation of vascular tone and oxidative stress. NO directly inhibits infiltration of inflammatory cells and release of inflammatory cytokines [83]. eNOS activity and NO release are mainly regulated by post-translational modifications via fatty acid and phosphorylation as well as protein-protein interaction with other effector molecules including heat shock protein (HSP) 90 and Cav-1. Cav-1 binding to eNOS in the basal state suppresses eNOS dissociation from Cav-1 and synthesis of NO [84]. Thus, Cav-1 has a negative role as an allosteric modulators of eNOS [85]. Studies using Cav-1 scaffolding domain fused to the cellular internalization peptide and antennapedia (AP) showed that the amino acids 82–101 of Cav-1 contain the binding sites for eNOS and that F92 is the key residue for this inhibitory effect. Ca^2+^ regulation and signaling can also be involved in inflammatory responses. Cav-1 in combination with calcium could maintain the non-activated state of eNOS. When the concentration of intracellular Ca^2+^ increases, Ca^2+^ binds to the calcium regulatory protein, which results in replacement of Cav-1, and then eNOS is isolated from Cav-1, binds to HSP90 [86]. After depalmitoylation and phosphorylation of eNOS, the production of NO is increased; then palmitoylated eNOS returns to caveolae [87]. When Cav-1 expression is silenced by siRNA, eNOS activity and NO release are increased. Maniatis and colleagues [57] found that endocytosis of caveolae mediates the activation of eNOS. When endothelial cells are activated by glycoprotein gp60 (Glycoprotein60), Cav-1 regulates the endocytosis of caveolae and the production of NO. The augment of NO was occurred in 2 min and lasted for 20 min, which was accompanied by the Src kinase activation. The activation of the Src kinase can promote phosphorylation of Cav-1, Akt and eNOS, resulting in eNOS dissociation from Cav-1. The inhibitor of the Src kinase can prevent the production of NO. Cav-1 sequesters p42/44 MAPK cascade members including epidermal growth factor (EGF) receptor, Raf, microtubule-associated protein (MAP)-1 and extracellular signal-regulated kinase 1/2 (ERK1/2), resulting in inhibition of this pathway [88]. eNOS, on the other hand, might undergo phosphorylation. Folic acid promotes eNOS dephosphorylation at negative regulatory sites and increases phosphorylation at positive regulatory sites, resulting in a higher concentration of cGMP and phosphoinositide 3-kinase (PI3K)/Akt activity [89]. It was found that ABCA1 and ABCG1 can preserve eNOS activity in high-fat and high-cholesterol diet-fed mice. Increased interaction of Cav-1 with eNOS is also detected in aortic homogenates of high-cholesterol diet–fed ABCG1 deficient mice, which is accompanied by a decrease in eNOS activity and an impaired endothelial function [90]. LDL cholesterol-loaded ECs are known to increase the Cav-1/eNOS interaction with a decreased production of NO [91]. The enhancement of cholesterol efflux via ABCG1 reduces the inhibitory interaction between eNOS and Cav-1 [57].

Muhammad and colleagues demonstrate that Cav-1 deficiency dampens TLR4 signaling through eNOS activation [92]. Inhibition of eNOS results in a decreased production of pro-inflammatory cytokines, and then reduces the NF-κB activation in response to LPS in Cav-1 knockout mice. LPS induces TLR binding with MyD88 and initiate either MyD88-dependent or independent pathway [93,94]. MyD recruits IRAK4 (IL-4 receptor-associated kinase) and IRAK1 (IL-1 receptor-associated kinase). The kinase activity of IRAK4 is critical for the TLR-mediated immune response. The IRAK4 kinase-inactive-knock-in mice are resistant to TLR4 signaling-induced shock response due to impairment of TLR-mediated induction of pro-inflammatory cytokines and chemokines. IRAK1 is phosphorylated by IRAK4, leading to TRAF6 recruitment to receptor complex, and activation of TAK1, one of the members of MAPK family. TAK1 then activates NF-κB [95,96]. The levels of TNFα and macrophage inflammatory protein (MIP) induced by LPS were significantly decreased in plasma of Cav-1 null mice. Thus, eNOS activation secondary to Cav-1 deficiency induces IRAK4 nitration and subsequent impairment of its kinase activity, and thereby selectively dampens the TLR signaling mediated by the MyD88-IRAK4 pathway [97].

Ischemic preconditioning (IPC) resulted in the significant increase of NO/*S*-nitrosylation (SNO) signaling which derived from Cav-3-associated eNOS in hearts [98,99]. Profilin-1 reduced sarcolemmal caveolae, Cav-3 protein and inhibited eNOS/NO signal including eNOS activity and production of NO [100]. Considering that Cav-3 is expressed in the heart, its regulation could indirectly alter AS outcome.

### 4.3. Caveolae/Cav-1 and the COX Signaling Pathway

COX-2 has been considered a marker and a mediator in endovascular inflammation and cardiovascular disorders. It has been reported [101] that COX-2 is localized in the endoplasmic reticulum (ER) and nuclear envelope (NE) and caveolae-like structures. COX-2 induced by Phorbol-12-myristate-13-acetate (PMA) or interleukin-1β (IL-1β) co-localizes with Cav-1 to caveolae and forms a complex with Cav-1. COX-2 binding to Cav-1 in ER promotes the degradation of COX-2. The level of COX-2 expression is higher in Cav-1 deficient mice than that in the wild-type mice. RNAi knockdown of Cav-1 increases the COX-2 protein level and decreases the accumulation of the ubiquitinated COX-2. In addition, deletion of the carboxy (C)-terminus of COX-2, which contains a unique 19-amino acid segment compared with COX-1, results in a reduction of Cav-1 binding capacity and attenuation of COX-2 degradation [102,103]. These data suggest the Cav-1 regulates inflammation via modulating COX-2.

### 4.4. Caveolae/Cav-1 and the MAPK Signal Pathway

The MAPK and phosphatidylinositol 3 kinase (PI3K) pathways are activated by many factors including LPS and intimately linked with the regulation of cytokine production [102]. Both MAPK and PI3K are found to localize in caveolae [104], suggesting that the MAPK and PI3K signaling cascades can be attractive target candidates for Cav-1 inhibition. Cav-1 augments p38 phosphorylation, but inhibits phosphorylation of c-Jun N-terminal kinase (JNK), ERK1/2 and Akt. The p38 inhibitor SB203580 blocks the effect of Cav-1 on LPS-induced cytokine production. NF-κB and the activator protein 1 (AP-1) are two well-known transcriptional factors that regulate LPS induction of cytokine production, while p38 suppresses the activities of NF-κB and AP-1. Therefore, Cav-1 confers anti-inflammatory effect in macrophages via the MKK3/p38 MAPK pathway. An increase in LPS-mediated activation of p38 and a decrease in activation of JNK, NF-κB and AP-1 by Cav-1 lead to the protection against inflammation [105]. Silencing of Cav-1 or depletion of cholesterol suppresses IL-1β-induced p38-MAPK and dual specificity mitogen-activated protein kinase kinase 2 (MEK2) activation and reduces tube formation and migration of ECs [9].

In early endoplasmic reticulum stress, expression of Cav-1 is increased, but decreased with prolonged treatment with thapsigargin or its high concentration, which is also related to p38/MAPK pathway [106].

### 4.5. Caveolae/Cav-1 and the Integrin/Adhesion Molecule Signaling Pathway

Adhesion molecules are the hallmarks of migration of inflammatory cells to the vascular intima and their activation, while integrin mediates cell adhesion [107]. Caveolae and Cav-1 are involved in integrin signaling pathway. Immunoprecipitation assay has confirmed the relationship between Cav-1 and integrin. Salani and colleagues recently found that Cav-1 is essential for integrin β1 located in caveolae. Phosphorylation of Cav-1 on Tyr14 is involved in integrin-regulated caveolae trafficking and signaling at focal adhesions in migrating cells [108]. Integrin-linked kinase acts as a scaffold to allow actin-microtubule interactions and proper positioning of Cav-1 close to the plasma membrane [109]. Treatment of HUVECs with cholesterol enhances the LPS-induced monocyte adhesion. Using isolated caveolae-enriched membranes, the studies showed that CAMs, including ICAM-1 and VCAM-1, co-localize with Cav-1 in caveolae. LPS up-regulates CAM expression and increases their co-localization. Exposure to cholesterol decreases the levels of CAMs in the caveolae. Co-immunoprecipitation and confocal microscopy assays further revealed that ICAM-1 interacts with Cav-1. Cholesterol treatment decreased this interaction and drove ICAM-1 out of caveolae. Knockdown of Cav-1 reduces the synergistic effects of cholesterol and inflammation [8]. These observations suggest the occurrence of the synergistic effects of hypercholesterolemia and inflammation in atherogenesis, and the caveolae/Cav-1 are involved in integrin/adhesion molecule signaling pathway.

### 4.6. Caveolae/Cav-1 and PPARγ and NF-κB Signal Pathways

PPARγ and RXR promote the expression of Cav-1, SR-B1, ABCA1, ABC-G1, cluster of differentiation 36 (CD36), apo-E, and LXR, which are involved in RCT [2]. PPARγ also plays pivotal roles in inflammation. Activation of PPARγ and/or LXRα interferes with activities of NF-κB, Signal transducer and activator of transcription (STAT), and AP-1 in macrophages. PPARγ reduces MMP-9 activity and inhibits the expression of IL-1β, IL-6 and TNF-α [110,111,112]. In murine macrophages, PPARγ activation represses the induction of several inflammatory response genes induced by LPS and IFN-γ, such as iNOS, COX-2, and IL-12 [113]. Accumulating evidence points to an intranuclear crosstalk between PPARγ and transcriptional factors on the promoters of inflammatory genes, a phenomenon known as transrepression [114]: PPARγ and transcription factors, such as NF-κB, AP-1, CCAAT-enhancer-binding proteins (C/EBP), and STAT, bind each other via protein-protein interaction and thus modulate their transcriptional activities [115].

Cav-1 binds TLR4, eNOS, MAPK and COX-2, respectively, to initiate different signal pathways, leading to inhibition of NF-κB transcriptional activity that blocks the expression of pro-inflammatory genes, such as TNF-α and IL-6.

## 5. Perspective and Conclusions

**Figure 1:**
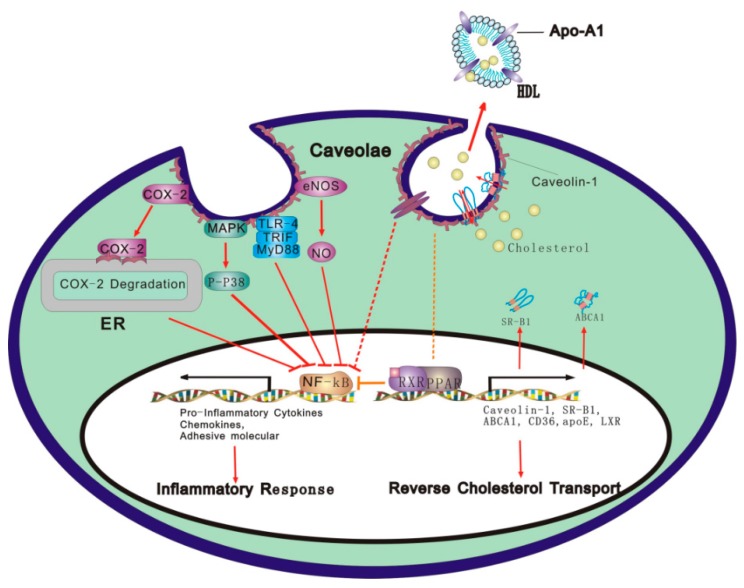
The primary mechanisms whereby caveolae/Cav-1 exerts its protective effects on inflammatory responses triggered by LPS or cytokines. On the one hand, Cav-1 binds to TLR4, eNOS, MAPK and COX-2, respectively, and initiates different signal pathways, resulting in the inhibition of NF-κB transcriptional activity that blocks the expression of pro-inflammatory genes, such as TNF-α and IL-6. On the other hand, the cholesterol-export activity of Cav-1 may account for its potent anti-inflammation properties. PPARγ may induce Cav-1 expression, enhance cholesterol efflux, and inhibit NF-κB transcriptional activity, further suppressing LPS-induced inflammatory response. Red arrows: direct promotion or leading to subsequent result; dished lines: indirect inhibition.

A variety of studies *in vivo* and *in vitro* have revealed the relationship between AS and RCT. Caveolae/Cav-1 is a center stage of cholesterol transportation during the period of RCT. Most recent studies show that inflammation impairs RCT, suggesting a new research direction to molecular mechanisms underlying the effect of inflammation on the development of AS. Inflammation could induce the formation of foam cells by disruption of cellular lipid metabolism, consequently leading to the AS (or plaque formation); conversely, dyslipidemias eventually result in inflammatory response. Therefore, lipid metabolism disorder is a prerequisite for the formation of AS, and inflammation is the sufficient condition for AS development. Both of them interact with each other. However, the molecular mechanisms of RCT and inflammation have not been well understood. As summarized in this review, previous studies and results in our group have demonstrated that caveolae/Cav-1 is a possible link between RCT and inflammation. We further speculate that caveolae/Cav-1 integrates with RCT and inflammation through a mechanism involving PPARγ and TLR-mediated inflammatory response (see Figure 1). More studies are warranted to explore the major determinants of this interplay’s response, and further attention should be given to the cross-talk signal transduction pathways among caveolae/Cav-1, inflammation and RCT.
